# Supplementary material for: Gas hydrate dissociation off Svalbard induced by isostatic rebound rather than global warming
Source: Nat Commun. 2018 Jan 8;9:83. doi: 10.1038/s41467-017-02550-9 (PMC5758787; doi:10.1038/s41467-017-02550-9)
Supplement: Supplementary file 1 — Supplementary Information [file 41467_2017_2550_MOESM1_ESM.pdf]

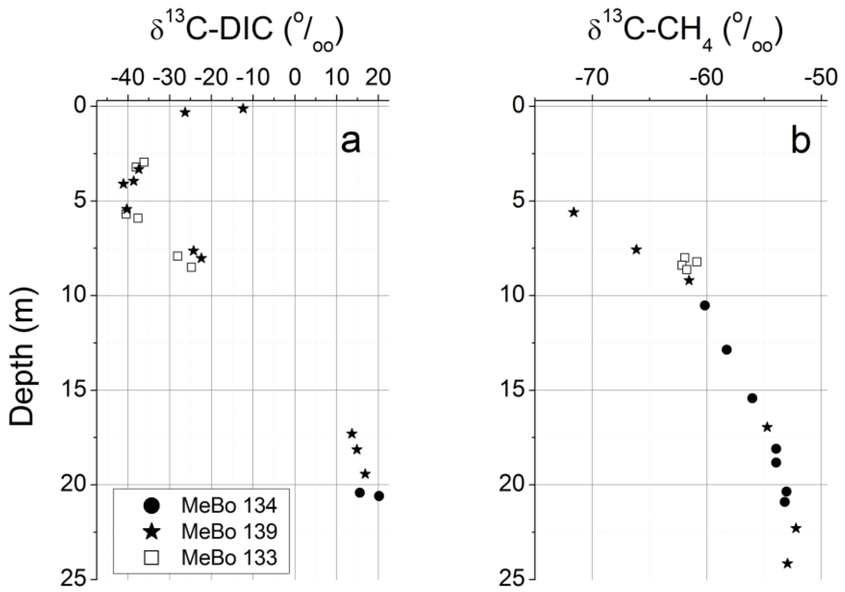

Supplementary Figure 1. Stable carbon isotope data at 391 m water depth (in $^{o}/_{oo}$ with respect to PDB standard). **(a)** δ^{13}C values of dissolved inorganic carbon (DIC). **(b)** δ^{13}C values of dissolved methane.

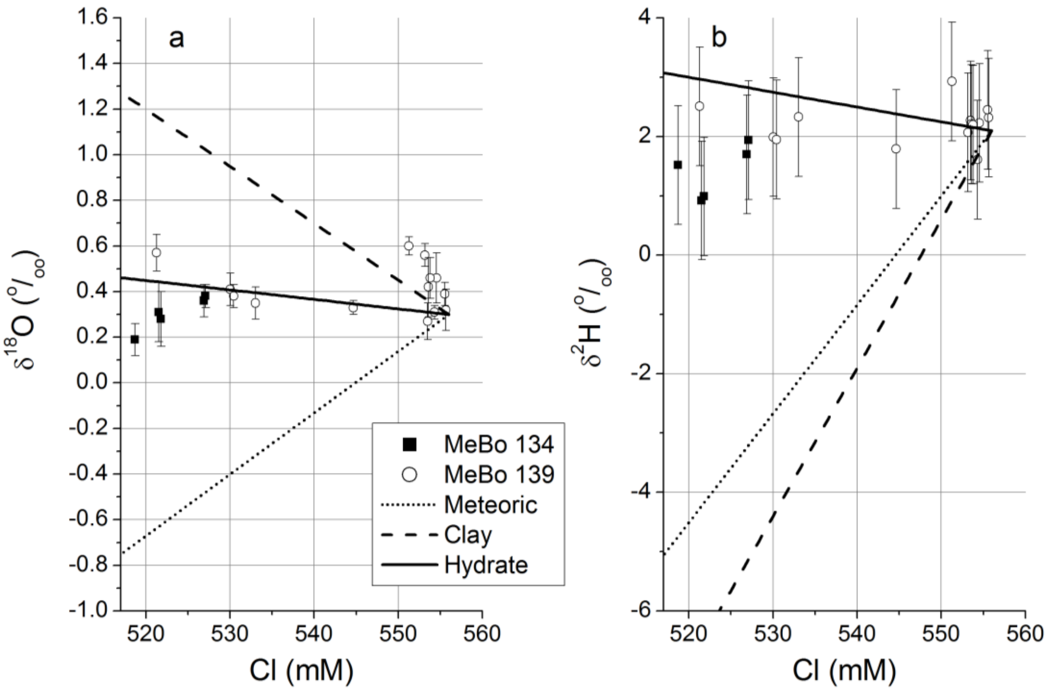

Supplementary Figure 2. Isotopic composition of pore fluids versus dissolved chloride concentration at 391 m water depth. Lines are calculated by mixing ambient seawater with freshwater from different sources including meltwater from northwest Svalbard (meteoric), water released form clay minerals during sediment diagenesis (clay), and hydrate water released upon gas hydrate dissociation (hydrate). **(a)** δ^{18}O values of pore water with respect to Vienna Standard Mean Ocean Water (VSMOW). **(b)** δ^{2}H values of pore water with respect to VSMOW.

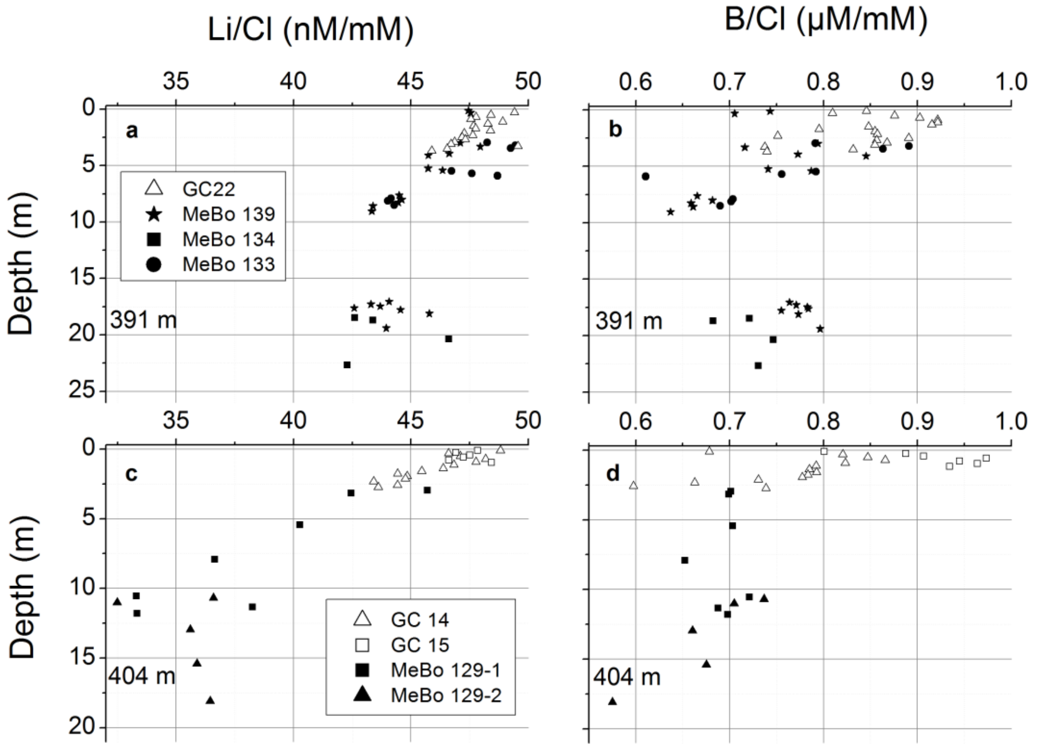

Supplementary Figure 3. Molar Li/Cl and B/Cl ratios in pore fluids. **(a)** Li/Cl at 391 m water depth. **(b)** B/Cl at 391 m water depth. **(c)** Li/Cl at 404 m water depth. **(d)** B/Cl at 404 m water depth.

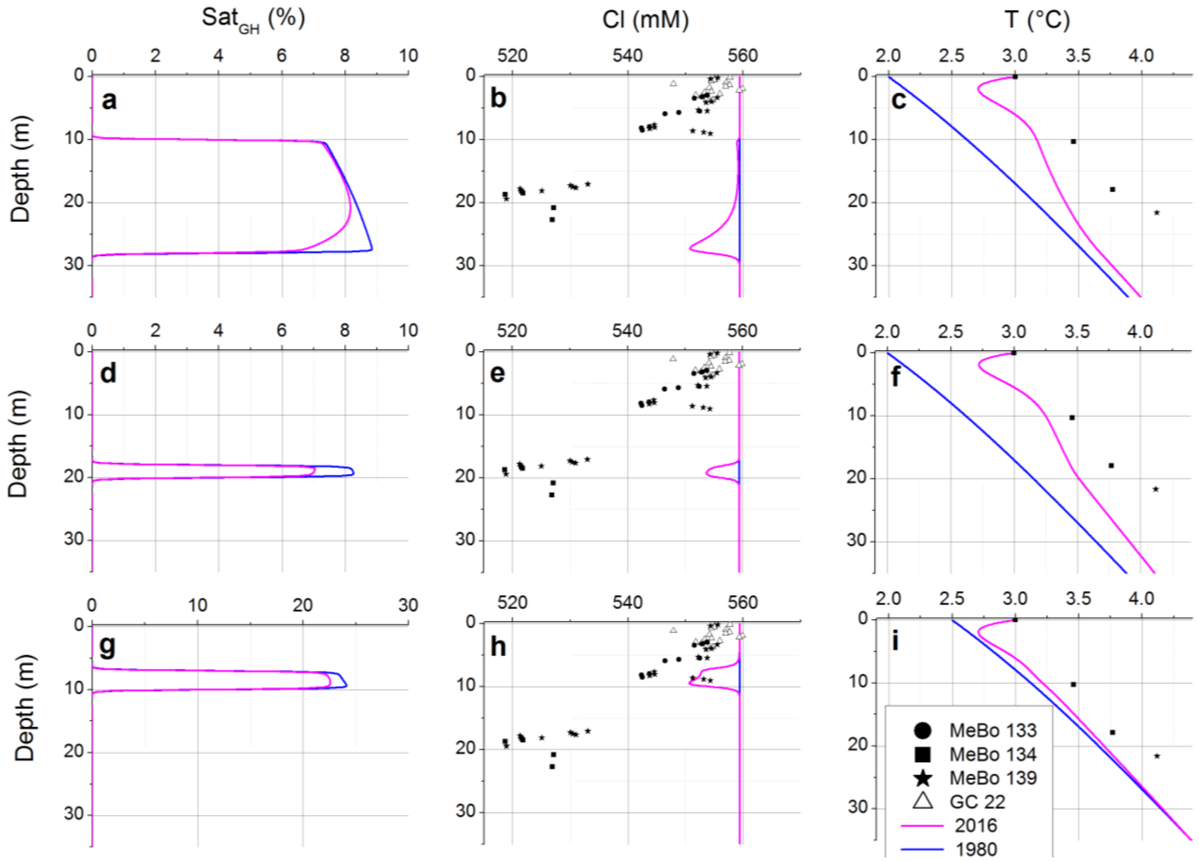

Supplementary Figure 4. Model results for hydrate melting at 391 m water depth. The first row **(a**, **b**, **c)** shows the initial values at 1980 and final results for 2016 of the simulation depicted in Fig. 4. The second row **(d, e, f)** shows the corresponding results for a different choice of the initial hydrate depth profile. The third row **(g, h, i)** depicts simulation results for elevated initial temperatures and hydrate saturations.

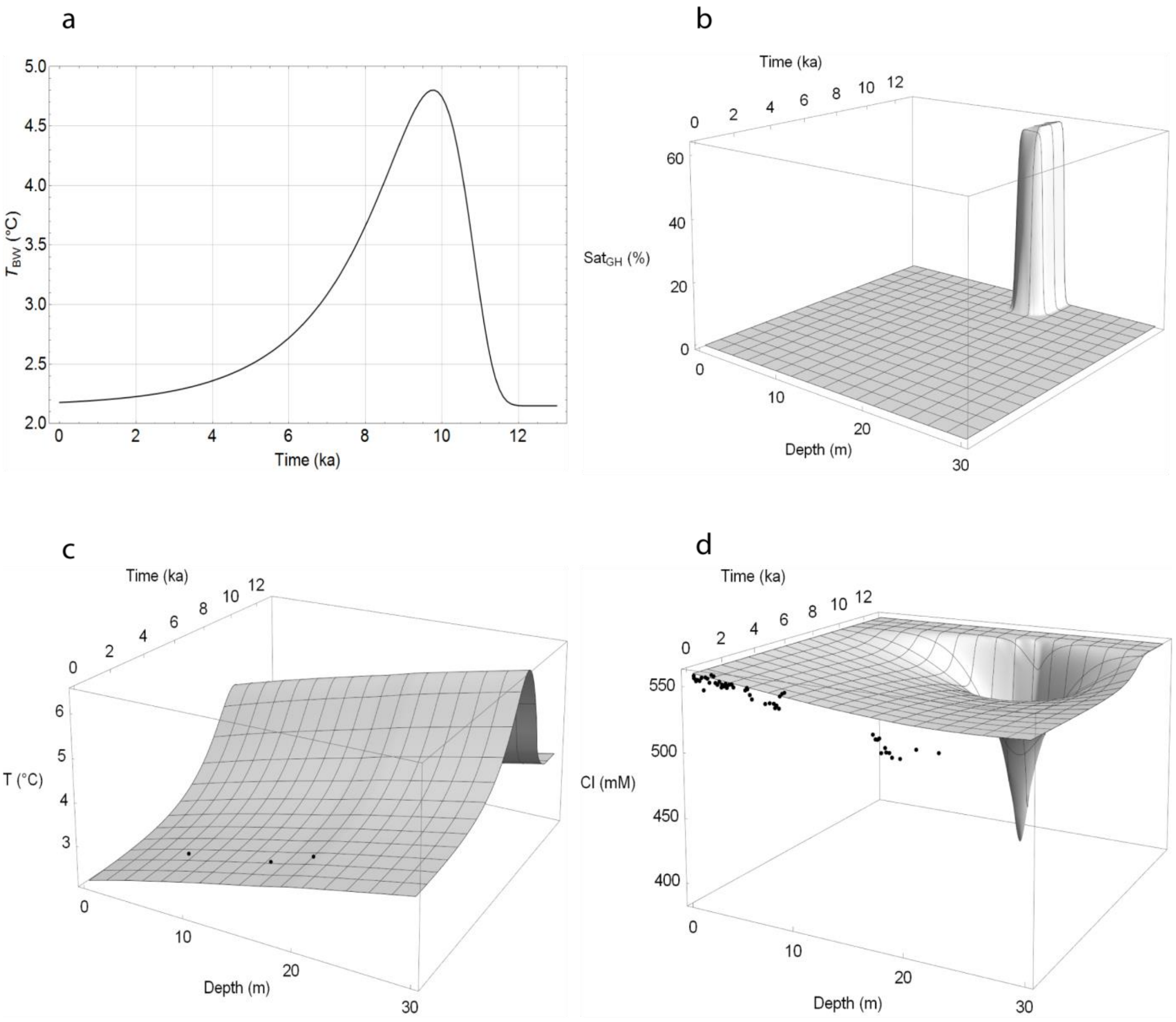

Supplementary Figure 5. Model results for Holocene hydrate melting at 391 m water depth. **(a)** Bottom water temperatures (T_{BW}) applied as model forcing. **(b)** Percent of pore space occupied by gas hydrate (Sat_{GH}). **(c)** Bulk sediment temperature (T). Dots indicate temperatures measured in drill holes at 391 m water depth (s. Fig. 2). **(d)** Dissolved chloride concentration in pore fluids (Cl). Dots indicate concentrations in cores retrieved at 391 m water depth (s. Fig. 2).

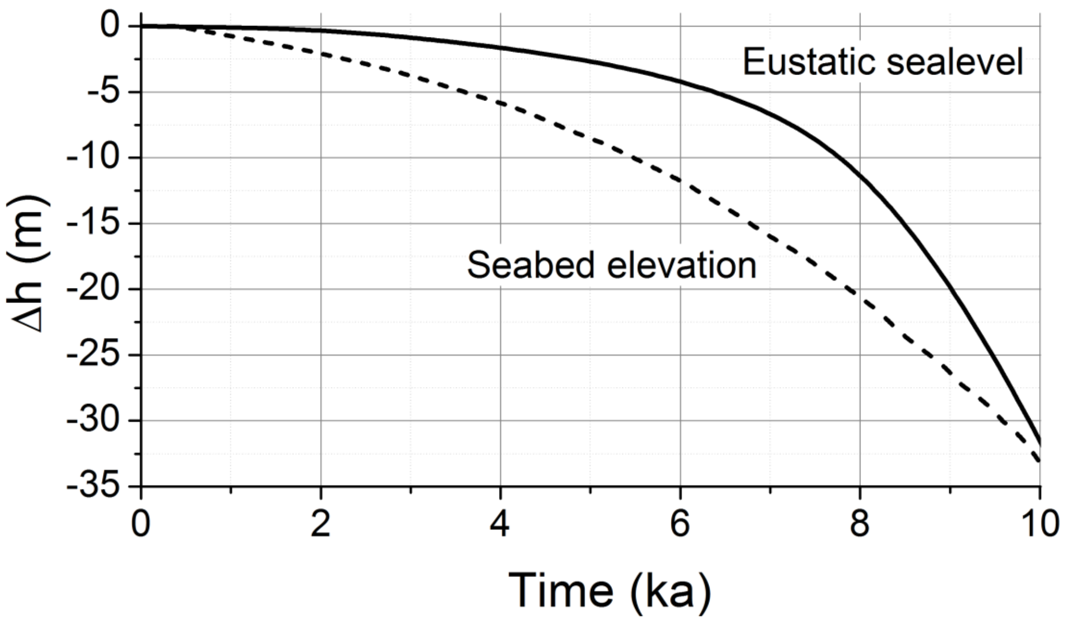

Supplementary Figure 6. Eustatic sealevel (solid line) [1] and change in seabed elevation at the upper continental slope of northwestern Svalbard as calculated with the ice sheet model (broken line). Relative sea level change is calculated as difference between eustatic sealevel and seabed elevation.

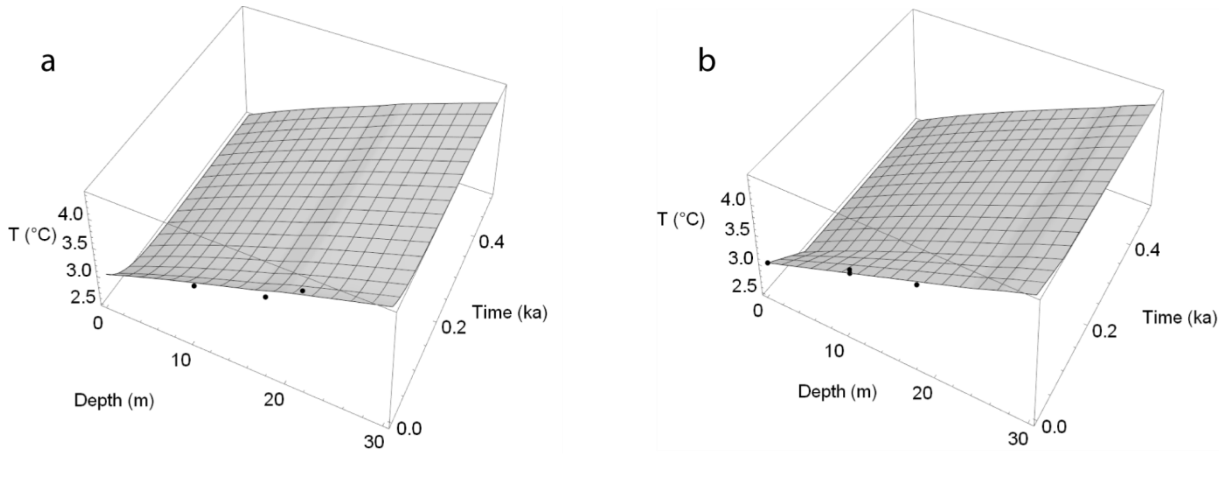

Supplementary Figure 7. Model results for sediment temperature. **(a)** 391 m water depth. **(b)** 404 m water depth. The bottom water temperature was maintained at 2.5°C for 8 ka – 0.1 ka and was enhanced to 3°C over the last 100 years of the model period. Temperature gradients of 45°C km^{-1} (391 m) and 50°C km^{-1} (404 m) were applied at the base of the model column (100 mbsf). Dots indicate data in cores retrieved at 391 m and 404 m water depth. Final temperature profiles at 0 ka are also shown in Fig. 2.

Supplementary Table 1: Location of the coring and drilling sites

GeoB Station No.	Station No. MSM57	Latitude (N)	Longitude (E)	Water depth (m)
GeoB21644-1	GC-22	78°33.132'	09°29.653'	391
GeoB21632-1	MeBo133	78°33.132'	09°29.653'	391
GeoB21632-2	MeBo134	78°33.132'	09°29.653'	391
GeoB21639-1	MeBo139	78°33.132'	09°29.653'	391
GeoB21631-2	GC-14	78°33.237'	09°27.336'	404
GeoB21631-5	GC-15	78°33.236'	09°27.337'	404
GeoB21626-1	MeBo129-1	78°33.212'	09°27.070'	404
GeoB21626-2	MeBo129-2	78°33.212'	09°27.070'	404

Supplementary Discussion

The freshwater component in Cl-depleted pore fluids could be meteoric water originating from Svalbard, water released from smectite and other water-bearing mineral phases during sediment diagenesis, a salt-depleted fluid created during hydrothermal phase separation, or water released during the dissociation of gas hydrates[2]. The stable isotopic composition of pore water ($\delta^{18}O$, $\delta^{2}H$) indicates that the freshwater component is neither of meteoric origin nor produced by the dewatering of smectite and other clay minerals (Supplementary Figure 2). The most plausible meteoric source would be glacial meltwater from northwestern Svalbard with a mean $\delta^{18}O_m$ value of -14.7 $^o/_{oo}$[3] and a $\delta^{2}H_m$ value of about -100 $^o/_{oo}$ as derived from the local meteoric water line[4]. Clay minerals formed by weathering processes on Svalbard in equilibrium with the depleted meltwater should contain chemically bound water enriched in $\delta^{18}O$ and further depleted in $\delta^{2}H$[5] with a composition of $\delta^{18}O_c$ = +14.2 $^o/_{oo}$ and $\delta^{2}H_c$ = -137 $^o/_{oo}$[6]. Mixing between these freshwater endmembers (meteoric, clay-derived) and near surface porewater (Cl = 556 mM, $\delta^{18}O_{sw}$ = 0.3 $^o/_{oo}$, $\delta^{2}H_{sw}$ = 2.1 $^o/_{oo}$) yields isotopic compositions for the Cl-depleted fluids that strongly deviate from our data (Supplementary Figure 2). Dissolved Li and B are depleted in all porewater samples with respect to seawater (Supplementary Figure 3). Li and B are removed from pore fluids at low temperatures[7, 8] and released into the pore water when sediments are heated to more than about 60°C[9]. Since the depleted dissolved B and Li values suggest a low-temperature origin of the freshened fluids, the freshwater does not originate from hydrothermal phase separation and mineral dewatering processes such as smectite–illite transformation that typically occurs at >60°C[10].

Since all other freshwater sources can be excluded, we conclude that the chloride depletion is caused by in-situ gas hydrate dissociation. Gas hydrates are enriched in both ^{18}O and ^{2}H with respect to ambient porewater[11]. At our sites, the water bound in methane hydrate should have isotopic compositions of $\delta^{18}O_h$ = 2.6 – 3.5 $^o/_{oo}$ and $\delta^{2}H_h$ = 16 – 24 $^o/_{oo}$[11]. Dissociation of gas hydrates with $\delta^{18}O_h$ = 2.6 $^o/_{oo}$ and $\delta^{2}H_h$ = 16$^o/_{oo}$ yields a mixing line that is broadly consistent with the data (Supplementary Figure 2). However, most samples tend to plot below the hydrate line even though we applied minimum estimates for hydrate isotope values. These second order deviations may be explained by the precipitation of ^{18}O-enriched carbonates and the microbial oxidation of organic matter and methane that releases ^{2}H-depleted water into the pore space[2]. It is also possible that water cages of the dissociating gas hydrates were not completely occupied by gas molecules and therefore even less enriched in ^{18}O and ^{2}H than assumed in our calculation[12]. Finally, the low isotopic fractionation observed in the Cl-depleted pore fluids may be explained by diffusion. The molecular

diffusion coefficient of water is higher than that of Cl and Na[13] such that $\delta^{18}O$ and $\delta^{2}H$ enrichments may relax more rapidly to background values than chloride and salinity depletions.

Supplementary References

1. Waelbroeck C, *et al.* Sea-level and deep water temperature changes derived from benthic foraminifera isotopic records. *Quaternary Science Reviews* **21**, 295-305 (2002).

2. Dählmann A, de Lange GJ. Fluid-sediment interactions at Eastern Mediterranean mud volcanoes: a stable isotope study from ODP Leg 160. *Earth and Planetary Science Letters* **212**, 377-391 (2003).

3. MacLachlan SE, Cottier FR, Austin WEN, Howe JA. The salinity: delta O-18 water relationship in Kongsfjorden, western Spitsbergen. *Polar Research* **26**, 160-167 (2007).

4. Yde JC, *et al.* Chemical and isotopic characteristics of a glacier-derived naled in front of Austre Gronfjordbreen, Svalbard. *Polar Research* **31**, 15 (2012).

5. Savin SM, Hsieh JCC. The hydrogen and oxygen isotope geochemistry of pedogenic clay minerals: principles and theoretical background. *Geoderma* **82**, 227-253 (1998).

6. Sheppard SMF, Gilg HA. Stable isotope geochemistry of clay minerals. *Clay Minerals* **31**, 1-24 (1996).

7. You C-Y, Spivack AJ, Gieskes JM, Rosenbauer R, Bischoff JL. Experimental study of boron geochemistry: Implications for fluid processes in subduction zones. *Geochimica et Cosmochimica Acta* **59**, 2435-2442 (1995).

8. Scholz F, *et al.* Lithium isotope geochemistry of marine pore waters - Insights from cold seep fluids. *Geochimica et Cosmochimica Acta* **74**, 3459-3475 (2010).

9. You CF, Castillo PR, Gieskes JM, Chan LH, Spivack AJ. Trace element behavior in hydrothermal experiments: Implications for fluid processes at shallow depths in subduction zones. *Earth and Planetary Science Letters* **140**, 41-52 (1996).

10. Hensen C, Wallmann K, M S, Ranero CR, Suess E. Fluid expulsion related to mud extrusion off Costa Rica - a window to the subducting slab. *Geology* **32**, 201-204 (2004).

11. Maekawa T. Experimental study on isotopic fractionation in water during gas hydrate formation. *Geochemical Journal* **38**, 129-138 (2004).

12. Tomaru H, Torres ME, Matsumoto R, Borowski WS. Effect of massive gas hydrate formation on the water isotopic fractionation of the gas hydrate system at Hydrate Ridge, Cascadia margin, offshore Oregon. *Geochemistry Geophysics Geosystems* **7**, doi:10.1029/2005GC001207 (2006).

13. Boudreau BP. *Diagenetic Models and Their Implementation*. Springer-Verlag (1997).
